# Ozone Induced Stomatal Regulations, MAPK and Phytohormone Signaling in Plants

**DOI:** 10.3390/ijms22126304

**Published:** 2021-06-11

**Authors:** Md. Mahadi Hasan, Md. Atikur Rahman, Milan Skalicky, Nadiyah M. Alabdallah, Muhammad Waseem, Mohammad Shah Jahan, Golam Jalal Ahammed, Mohamed M. El-Mogy, Ahmed Abou El-Yazied, Mohamed F. M. Ibrahim, Xiang-Wen Fang

**Affiliations:** 1 State Key Laboratory of Grassland Agro-Ecosystems, School of Life Sciences, Lanzhou University, Lanzhou 730000, China; hasanmahadikau@gmail.com (M.M.H.); waseem17@lzu.edu.cn (M.W.); 2 Grassland and Forage Division, National Institute of Animal Science, Rural Development Administration, Cheonan 31000, Korea; atikbt@korea.kr; 3 Department of Botany and Plant Physiology, Faculty of Agrobiology, Food and Natural Resources, Czech University of Life Sciences Prague, 16500 Prague, Czech Republic; skalicky@af.czu.cz; 4 Department of Biology, College of Science, Imam Abdulrahman Bin Faisal University, Dammam 383, Saudi Arabia; nmalabdallah@iau.edu.sa; 5 Key Laboratory of Southern Vegetable Crop Genetic Improvement in Ministry of Agriculture, College of Horticulture, Nanjing Agricultural University, Nanjing 210095, China; shahjahansau@gmail.com; 6 Department of Horticulture, Sher-e-Bangla Agricultural University, Dhaka 1207, Bangladesh; 7 College of Horticulture and Plant Protection, Henan University of Science and Technology, Luoyang 471023, China; ahammed@haust.edu.cn; 8 Vegetable Crop Department, Faculty of Agriculture, Cairo University, Giza 12613, Egypt; elmogy@agr.cu.edu.eg; 9 Department of Horticulture, Faculty of Agriculture, Ain Shams University, Cairo 11566, Egypt; ahmed_abdelhafez2@agr.asu.edu.eg; 10 Department of Agricultural Botany, Faculty of Agriculture, Ain Shams University, Cairo 11566, Egypt; ibrahim_mfm@agr.asu.edu.eg

**Keywords:** guard cells, reactive oxygen species (ROS), hydrogen peroxide (H_2_O_2_), abscisic acid, ethylene, salicylic acid

## Abstract

Ozone (O_3_) is a gaseous environmental pollutant that can enter leaves through stomatal pores and cause damage to foliage. It can induce oxidative stress through the generation of reactive oxygen species (ROS) like hydrogen peroxide (H_2_O_2_) that can actively participate in stomatal closing or opening in plants. A number of phytohormones, including abscisic acid (ABA), ethylene (ET), salicylic acid (SA), and jasmonic acid (JA) are involved in stomatal regulation in plants. The effects of ozone on these phytohormones’ ability to regulate the guard cells of stomata have been little studied, however, and the goal of this paper is to explore and understand the effects of ozone on stomatal regulation through guard cell signaling by phytohormones. In this review, we updated the existing knowledge by considering several physiological mechanisms related to stomatal regulation after response to ozone. The collected information should deepen our understanding of the molecular pathways associated with response to ozone stress, in particular, how it influences stomatal regulation, mitogen-activated protein kinase (MAPK) activity, and phytohormone signaling. After summarizing the findings and noting the gaps in the literature, we present some ideas for future research on ozone stress in plants

## 1. Introduction

Ozone is formed in the troposphere through the action of sunlight-driven chemical reactions involving nitrogen oxides and volatile organic compounds [1]. Because of anthropogenic activities, tropospheric ozone concentrations have increased significantly, especially in industrial and urban areas where ozone air pollution negatively affects plant growth and physiology [2]. Ozone’s effect on a particular plant species depends on several factors including concentration and exposure time. Previous studies reported that low levels of ozone decreased photosynthetic and reproductive capacity, as well as accelerating senescence and abscission, and causing foliar damage [3]. Ozone pollution is ultimately responsible for huge economic losses from decreased forest productivity and crop yield [4]. An imbalance in ozone is also associated with global warming and climate change [5].

Plant exposure to ozone produces a number of specific changes in gene expression, metabolic profiles, and enzyme activities. In sensitive accession, acute O_3_ exposure resulted in increased cell death, lesion development, and decreased photosynthesis [2]. After initiation of ozone exposure, protein modifications can occur quickly, sometimes within just a few minutes [6], because ozone is actively engaged in the generation of ROS in the apoplast. Thus, the formation of apoplastic ROS by ozone can be used as a non-invasive research tool to activate ROS-responsive signaling pathways. The results obtained from ozone-induction experiments proved that the pollutant played a direct, active role in apoplastic ROS signaling [7]. As ozone is highly reactive and unstable, it can induce oxidative stress in plants by chemically modifying various molecules to produce short-lived ROS such as peroxides, hydroxyl radicals, and superoxide [8].

Studies on the effects of ozone on stomatal regulation by phytohormones are limited in the literature. Ozone activates different signaling pathways in plants which are integrated into complex regulatory systems involving phytohormones such as abscisic acid (ABA), salicylic acid (SA), jasmonic acid (JA), and ethylene (ET), ROS, and calcium as secondary messengers [9]. Although significant progress has been made during the past decades in understanding the ozone response mechanisms in guard cells, there are still many gaps in the research. Our review describes recent advances in understanding how ozone enters leaves via stomata, generates ROS, and alters the activity of mitogen-activated protein kinases (MAPKs) and phytohormones in signaling pathways, and the molecular mechanisms of leaf damage formation, NO production, and alteration of metabolite profiles.

## 2. Ozone Sources, Formation, Emission, and Transport

Ozone (O_3_) is found throughout the atmosphere but its concentration peaks in two regions, the troposphere (0–15 km) and the stratosphere (15–50 km). The ground-level region of the troposphere is the principal source of toxic air pollutants like ozone. As a result of increased global atmospheric temperatures, the interaction of ultraviolet radiation with primary tropospheric pollutants such as nitrogen oxides and volatile organic compounds, results in the formation of secondary air pollutants including ozone [10]. Studies of atmospheric chemistry have identified several complex processes that may be involved in the formation of tropospheric ozone. Ultraviolet radiation can cause photodissociation of nitrogen dioxide, NO_2_, resulting in the formation of nitrogen monoxide (NO, nitric oxide) and free oxygen atoms. These free oxygen atoms, in turn, react with O_2_ to form ozone, O_3_ (reactions (i)–(iv))

NO_2_ + O_2_ (hν) ↔ NO + O_3_ (i)

CO + 2O_2_ (hν) → CO_2_ + O_3_ (ii)

CH_4_ + 4O_2_ + 2hν → HCHO + H_2_O + O_3_ (iii)

RH + 4O_2_ + 2hν → R′CHO +H_2_O + O_3_ (iv)

In regions with a lower NO_2_/NO ratio, the reaction of O_3_ with NO can lead to the destruction of ozone. However, volatile organic compounds (VOCs) such as methane can cause oxidation of NO and increase the NO_2_/NO ratio [11], ultimately resulting in a shift in the chemical reaction towards O_3_ accumulation. In addition, the production of atmospheric ozone is strongly correlated with alterations in precursor concentrations, a relationship that highlights the non-linearity of the O_3_-VOCs-NOx system [12]. Plants produce a broad range of natural VOCs, and the continuation of global climate change may cause an escalation in VOC levels, which contributes to an increase in peak tropospheric ozone. The photolysis of this excess ozone could give rise to the increased radical formation that can react with carbon monoxide and organic species, leading to additional ozone production.

Most air pollutants, like ozone precursors, emerge from mid-latitude regions, where long-range transport of emissions is dominated by westerly winds from source to downwind regions at both local and global scales. The lifespan of free ozone in the troposphere ranges from weeks to months, which is compatible with long-range transport that happens on a timescale of days to weeks. The ozone in the middle and upper troposphere travels faster than the ozone in the lower troposphere, owing to the action of strong winds at high altitudes. When an ecosystem encounters circumstances like air pollution, it can stimulate ozone formation at a distance from the precursor source regions [13]. The effects of ozone pollution are clearly observed on leaves, but because of these potential transportation events, care must be exercised in identifying the source of the ozone.

## 3. Ozone-Induced Stomatal Regulation in Plants

### 3.1. Stomata Control the Influx of Ozone into Leaves

**Figure 1:**
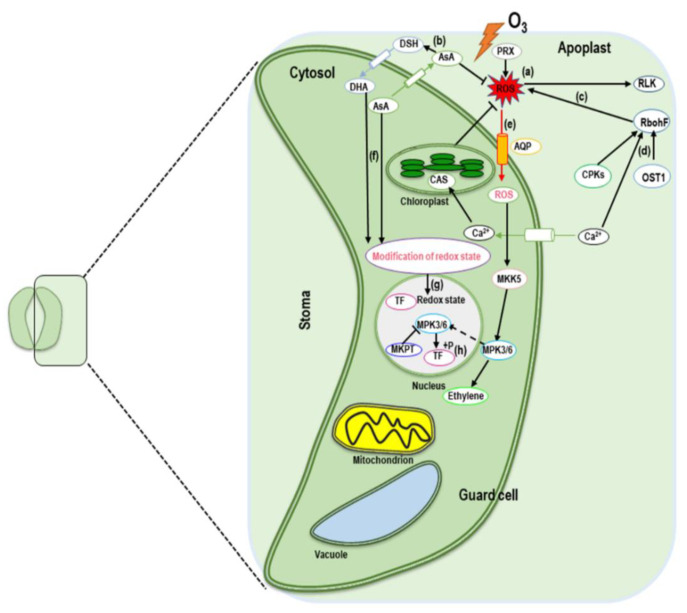
A proposed model associated with signaling in guard cells in response to ozone stress. Ozone leads to the formation of free radicals (ROS) in the apoplast and a rise in Ca^2+^ level in guard cells (a). Several apoplastic antioxidants including ascorbic acid (AsA) scavenge ROS and inhibit their generation (b). The mechanism of ROS defense triggered by high ozone concentrations involves two key antioxidants—peroxidases (PRX) and NADPH oxidases (RbohF) (c). RbohF in the signal transduction process is activated by second messengers including Ca^2+^, open stomata 1 (OST1), and calcium-dependent protein kinases (CPKs) (d). Apoplastic ROS may influence plasma membrane sensor proteins and receptor-like kinases, (RLKs) such that ROS (e.g., H_2_O_2_) can be transferred through aquaporin (AQP) channels (e). Cytosolic Ca^2+^ is sensed in chloroplasts by the calcium-sensing receptor (CAS). Apoplastic oxidized dehydroascorbate (DHA) and AsA move across the PM into the cytosol and regulate redox homeostasis (e), which is also sensed by redox-sensitive transcription factors (TFs). Triggering of mitogen-activated protein kinases 3/6 (MPK3/6) (f). Activation of MPK3/6 and translocation to the nucleus leads to phosphorylation and activates transcription factors (TFs) (g). Solid and broken lines indicate direct and indirect interactions, respectively.

Ozone can enter the leaves through the stomata with guard cells regulating the opening and closing. Stomatal apertures in leaves control the ozone flux in various ways. Past studies reported that ozone in the range of 50 to 75 ppb decreased stomatal conductance, which restricts the flow of CO_2_ into the leaves [14]. The reduction of stomatal conductance was observed for up to several hours from the beginning of ozone exposure. Recent studies reported that prior to the longer-lasting reduction in conductivity, ozone stress caused a rapid transient increase or decrease (RTI or RTD) in stomatal conductance [15]. The RTD can occur within a few minutes of ozone exposure, depending on its concentrations [7]. Guard cell ion channels and transporters are the key factors that regulate stomatal pore size, and they also control the transportation of osmotically active molecules across the plasma membrane and tonoplast [16]. Activation of guard cell ion channels is essential for stomatal closure and also crucial for limiting the influx of ozone into leaves. The rapidly deactivating R-type and the slowly deactivating S-type are the two principal types of anion channel involved in stomatal closure [17]. The S-type slow anion channel 1 (SLAC1) is vital for stomatal closure induced by various stimuli, including ozone, which was identified in screening an Arabidopsis mutant for ozone sensitivity [18]. Ozone is not the only factor inducing stomatal closure by SLAC1, however. It can also be triggered by ABA, NO, calcium, and light/dark transitions [17,18]. The protein kinase, open stomata 1 (OST1), activates SLAC1 by phosphorylating the N-terminal region of the ion channel [19] (Figure 1).

In addition to OST1, several calcium-dependent protein kinases (CPKs) and guard cell hydrogen peroxide-resistant 1 (GHR1) can also phosphorylate the N-terminal region of SLAC1 and this can be reversed by the PP2C phosphatases, ABA-insensitive 1 and 2 (ABI-1 and ABI-2) [7]. The ABA receptors, pyrabactin resistance 1 (PYR1) and PYR1-like (PYL), and the regulatory components of ABA receptors (RCAR) inhibit PP2C activity [20], which leads to anion efflux activation and stomatal closure by phosphorylation of SLAC1 [21]. ROS also inhibited ABI-1 and ABI-2 activity [22], indicating an ABA-independent pathway for OST1 activation. It has been confirmed that the above signaling pathways were essential for ozone-induced rapid stomatal closure [23]. In addition, ABI-1 can directly affect the activity of SLAC1 by dephosphorylating the SLAC1 N-terminal region [24]. It has been reported that SLAC1, or related anion channels, are involved in cryptogenic-induced ion fluxes in tobacco BY-2 (*Nicotiana tabacum* L. cv. Bright Yellow-2) cells in response to ozone [25].

Guard cell outward-rectifying K^+^ (GORK) channel activation is also important in stomatal regulation in response to ozone, which triggers membrane depolarization resulting in ozone-induced programmed cell death (PCD) [26]. Previous studies provided evidence that inhibition of the inward-rectifying K^+^ channel, KAT1, was crucial for stomatal closure [27]. OST1 phosphorylates KAT1 at the C-terminus, which interferes with anion channel activity leading to rapid stomatal closure [28]. Thus, we conclude that OST1 and SLAC1 are the major elements for stomatal closure in response to ozone.

Although OST1 plays a crucial role in ABA-dependent stomatal responses by controlling the ion channels, the ABA-independent stomatal responses through OST1 in the stomatal closure induced by ROS cannot be overlooked. Stomatal closure can be triggered by different factors, including ozone, which are supported by different pathways for the activation of SLAC1. It can be concluded that ROS plays a crucial role in stomatal closure, either directly or indirectly, by modulating several signaling pathways. This exemplifies the deep connection between ROS and ozone with regard to plant stress responses.

### 3.2. Stomatal Closure and Opening Is Linked to ABA and Ethylene and Shows Cross-Talk in Response to Ozone

**Figure 2:**
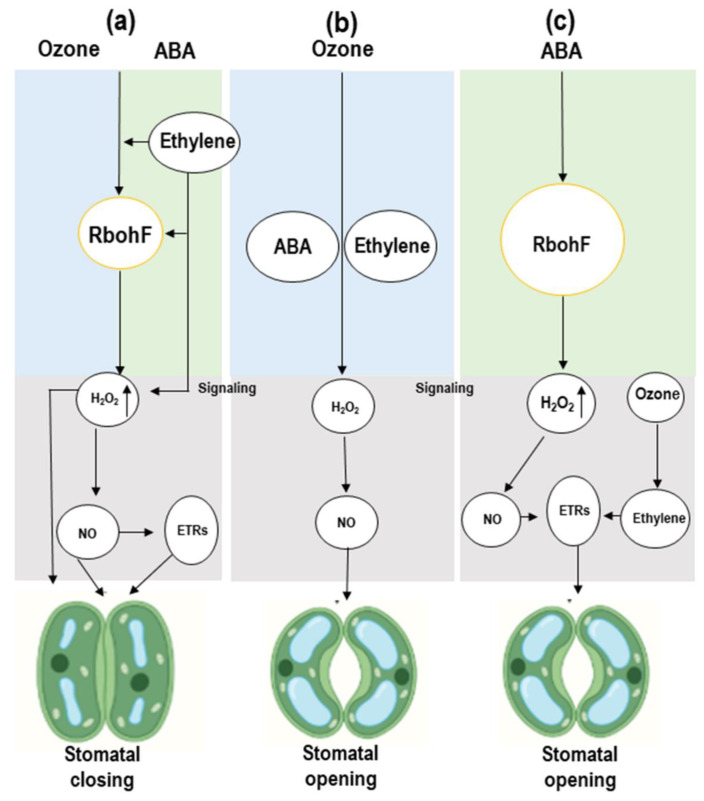
A schematic model of phytohormone-induced guard-cell signaling associated with stomatal regulation in response to ozone (O_3_), adapted from Wilkinson and Davies (2010) [31]. (**a**) Ozone and ethylene (ET) can substitute within the abscisic acid (ABA) signal transduction branch leading to stomatal closure through H_2_O_2_ in the absence of ABA. (**b**) A higher level of H_2_O_2_ and NO might prevent stomatal closure in the presence of ABA, O_3_, and ET. (**c**) ET acts via the ETR1 receptor to inhibit response to ABA-induced H_2_O_2_ and prevent stomatal closure in the presence of ABA.

Stomatal closure and opening is an important factor in plant growth regulation. ABA and ethylene (ET) are crucial phytohormones that have significant roles in stomatal regulation in plants [29]. It was confirmed by past studies, that these phytohormones directly or indirectly regulate stomatal opening and closing, but their effects on stomata in the presence of ozone need further study. It was observed that when plants with responsive stomata were exposed to ozone, foliar ABA levels increased quickly. Stomatal closure in response to ozone exposure results from the direct oxidation of ABA precursors resulting to ABA synthesis, although in normal photosynthesis, the significance of this ROS interaction is unknown [30]. ABA can increase H_2_O_2_ production through NADPH oxidases such as AtRbohF (Figure 2).

This H_2_O_2_ is an important signaling molecule that triggers NO production for stomatal closure in plants [32]. H_2_O_2_-dependent ABA signaling in guard cells requires the ethylene receptor, ETR1, downstream of H_2_O_2_, as H_2_O_2_ -mediated stomatal closure is disrupted in the ethylene-insensitive Arabidopsis mutant etr1-7. Stomatal ABA responses in the etr1-1 or ein3-1 ethylene-insensitive Arabidopsis mutants were not antagonized by ethylene [9]. ETR1 plays two roles in guard cells, one mediating ethylene sensing, and the other H_2_O_2_ signaling. It is possible that ethylene binding to ETR1 prevents ETR1 from performing its other role in signaling the presence of H_2_O_2_, whether it is generated through ozone or ABA, or through ethylene itself [33] (Figure 2). Ethylene may induce stomatal closure through substituting within the ABA signaling pathway. Given the above results and the ozone-induced H_2_O_2_ involvement in regulating stomata, it would suggest that ozone could cause stomatal movement in plants.

## 4. MAPK Cascade Signaling in Response to Ozone Stress

MAPK signaling is activated in response to various abiotic stresses [34,35]. The MAPK signaling mechanism includes three types of protein kinase, which activate and phosphorylate each other: MAPK kinase (MKK) activated by a MAPK kinase kinase (MEKK), which in turn, phosphorylates and activates a MAPK (MPK). These MAPK signaling units, MKK-MPK or MEKK-MKK-MPK, are involved in specific stress responses [36]. In response to ozone, the MAPKs, MPK3, and MPK6 were activated quickly and transiently (0.5–2 h) in two *Arabidopsis* strains. After activation, MPK3/6 translocated into the nucleus where they regulated the expression of the specific genes, SIPK and WIPK, commonly known as tobacco orthologs, which were found to be induced by ozone [37]. MPK3 and MPK6 were mutually regulated such that MPK3 inhibition led to further strong activation of MPK6 and MPK6 suppression resulted in prolonged and enhanced MPK3 activation [38]. In response to stress from plant pathogens, MKK4/MKK5 were activated by MEKK1 [39]. MKK5 was also involved in MPK3/MPK6 activation in response to ozone exposure [40]. A proper ozone response is required to mediate the optimal balance of MPK3/MPK6 activities because increases in activation time or any perturbations in the strength lead to increased ozone sensitivity. For example, the activation of MPK3/MPK6 in the ozone-sensitive radical-induced cell death1 *(rcd1)* mutant was prolonged compared to wild-type plants [41]. It has been reported that MPK3/MPK6 substrates are linked to ethylene biosynthesis and signaling, transcriptional factors, and nitric acid (NO) signaling [42] (Figure 2). Moreover, the MKK4/MKK5–MPK3/MPK6 signaling unit is crucial for stomatal regulation, which indirectly affects ozone entrance into leaves [43]. The question of specificity needs to be addressed to determine exactly how a few kinases can regulate all the varied stress responses.

## 5. Alterations of Phytohormone Signaling in Response to Ozone Stress

### 5.1. Ozone-Induced Regulation of Signaling Molecules (ROS, H_2_O_2_, and NO)

Nitric oxide (NO) is one of the key signaling molecules induced in plants in response to several stressors including O_3_ [44]. NO and H_2_O_2_ accumulation was observed in tobacco plants within 1.5 h of ozone exposure [45]. H_2_O_2_ can interact with NO, and NO donors such as sodium nitroprusside (SNP) can induce H_2_O_2_ accumulation in plants [46]. Guanosine 3, 5-cyclic monophosphate (cGMP) is a known second messenger of NO, and its formation was observed later at >2 h of ozone exposure [45]. In Arabidopsis, spatiotemporal analysis revealed that ozone-induced NO generation occurred first in the guard cells with a peak at 1.5 h of ozone exposure. After that, NO was observed in the palisade spongy mesophyll cells. When plants were exposed to 2 h ozone treatments, the NO donor SNP induced defense-related gene expression. Moreover, combined treatment with ozone and SNP downregulated defense-related gene expression and ethylene biosynthesis. These results indicated that ozone-induced hormone signaling was modulated by NO and that a certain NO level was essential for optimal ozone responses. NO reduction in an Atnoa1 mutant and overproduction of NO in a different Atnoa1 mutant correlated with ozone sensitivity. Cysteine residues of proteins were modified by NO (similar to ROS) via S-nitrosylation. Several proteins such as non-expresser of PR genes 1 (NPR1), RBOHD, and metacaspase 9 were modified by NO involved in ROS signaling (Huang et al., 2019). Both ROS and NO participate in the generation of reactive nitrogen species, which results in the formation of a signaling molecule known as 8-nitro-cGMP that ultimately induces stomatal closure in plants. Considering all these lines of evidence suggests an active role of signaling molecules like NO, H_2_O_2_, and ROS in the regulation of plant responses to ozone.

### 5.2. Phytohormones Involved in Signaling and Gene Expression during Ozone-Induced Oxidative Stress and Cellular Injury

A number of abiotic stressors including ozone cause physical, biomechanical, and molecular alterations in plants as a result of ROS generation, oxidative stress induction, cellular injury, and apoptosis. Phytohormones are recognized as important tools for mitigating the negative impact of abiotic stresses in plants. In the sub-sections, we focus on the involvement of several phytohormones in signaling and gene expression along with alleviation of ozone-induced oxidative stress and cellular injury in plants

#### 5.2.1. Salicylic Acid

Salicylic acid (SA) is a multifunctional phenolic compound involved in plant growth and development, along with responses to numerous stresses including ozone [47]. SA is known to be involved in the same signaling networks as other classical phytohormones such as jasmonic acid and ethylene in response to ozone. Furthermore, SA has been known to cause stomatal closure [48]. Together with other signaling, the guard cell SA signaling and its cross-talk with other signaling play critical roles in stomatal immunity [49], however the molecular mechanism remains unknown. SA is synthesized by the phenylpropanoid pathway or via isochorismate synthase in tobacco, tomato, and Arabidopsis [50]. Phenylalanine ammonia-lyase (PAL) is one of the enzymes that are crucial for SA biosynthesis in plants [51]. It has been reported that SA-accumulation because of ozone stress can be reduced by ET-mediated alteration of the expression of PAL and chorismate mutase (CM) in tobacco plants [52]. Arabidopsis ecotype Wassilewskija (WS) exposed to ozone showed lower expression of the AtSR/NFkB family of redox-sensitive transcription factors and the C2-domain proteins as well as other genes associated with cell wall growth and critical point drying (CPD), such as inactive poly[ADP-ribose] polymerase RCD1 (protein radical-induced cell death 1), which was recognized as a likely candidate gene [53]. 14C-labeled benzoic acid can be used as a precursor of SA in the phenylalanine pathway to measure SA synthesis, and it was observed that isochorismate synthase (ICS) mRNA expression was not induced in ozone-exposed tobacco and ICS activity was low [52]. In Arabidopsis, however, the ICS activity was increased in response to O_3_. The salicylic acid induction-deficient 2 (sid2) Arabidopsis mutant lacks ICS1 activity, and SA was found to be lower in the mutant in response to ozone [52]. These studies supported the conclusion that SA is synthesized through benzoic acid from phenylalanine in tobacco and through isochorismate in Arabidopsis.

It has been reported that SA accumulation was correlated with leaf lesion formation during ozone exposure to plants [47]. NahG gene expression inhibited SA accumulation and decreased lesion formation in the tobacco cultivar ‘Xanthi’ exposed to ozone. A mediated defense response was reported to be induced during O3 exposure in plants. In the ozone-tolerant Arabidopsis Col-0 ecotype, a NahG-transformed line exhibited higher O_3_ sensitivity compared to Col-0 plants [54]. Ozone-inducible SA has a dual function depending on its level of production. An Arabidopsis Cvi-0 ecotype known for ozone sensitivity accumulated more than three times the amount of SA than an ozone-tolerant Col-0 ecotype [54]. Exposure to ozone enhanced PR1 (AT2G14610) expression in Cvi-0 from a control stage that was still greater than those in Col-0 (Table 1) [53].

Ozone-mediated changes in the pathway involving AtSR, a homolog of the mammalian NFkB family of redox-sensitive transcription factors, resulted in changes in chaperones, WRKY, and C_2_H_2_ proteins and antioxidants [53]. It has also been documented that the generation of antioxidants such as glutathione reductase, ascorbate peroxidase, and glutathione peroxidase were induced in response to ozone in Col-0 plants [54]. Table 2 shows the responses of antioxidant genes to ozone and the controls.

Furthermore, O_3_-induced regulation of genes has been reported to wild-type and several mutants of model plants Arabidopsis (Table 3).

However, this section can be summarized that SA is required to maintain the cellular redox state and antioxidant defense responses under ozone stress in plants, as well as O_3_, which can involve directly in stress-induced candidate gene expression in model plants.

#### 5.2.2. Ethylene

Ethylene (ET) is one of the most important phytohormones involved in plant growth and development, and ET production is stimulated by plant exposure to a number of biotic and abiotic stresses [56]. Although the relationship between ET and abiotic stress alleviation is well known, in this section we discuss the functional response to ozone exposure in terms of the ET-responsive biosynthetic genes. The two key enzymes in ethylene biosynthesis, are ACC synthase (ACS) and ACC oxidase (ACO). S-adenosyl-L-methionine is converted to 1- aminocyclopropane -1-carboxylic acid (ACC), which undergoes oxidative cleavage by ACO to produce ethylene [57]. Both ACC synthase (ACS) and ACC oxidase (ACO) are encoded by similar gene families in a number of different organisms all known to catalyze the same reactions in ethylene biosynthesis [58]. Under ozone exposure in Arabidopsis, Cvi-0 showed increased expression of three known ET sensitive genes (AT1G49830, AT1G55150, and AT2G22300) with unknown functions (Table 1). The expression of At5g44440 and At2g26020 were reduced in both Cvi-0 and Col-0 during ozone exposure (Table 1) [53]. Expression of ST-ACS4 and ST-ACS5 is enhanced in potato, whereas LE-ACS1A, LE-ACS2, and LE-ACS6 are induced in ozone-exposed tomato plants [59]. The only major gene among the nine ACS genes [60], that triggers the evolution of ethylene when Arabidopsis is exposed to ozone. The amount of ethylene produced in response to ozone induction showed a correlation between the ozone-induced ethylene biosynthesis rate and the level of leaf injury in several species. When exposed to ozone, the ozone-tolerant ‘Bel-B’ tobacco generated less ethylene than the ozone-sensitive ‘Bel-W3′ strain [61]. Under ozone exposure, treatment with inhibitors of ethylene biosynthesis reduced leaf injury [61]. These findings suggest that the increase in ozone-induced leaf damage associated with ethylene might be due to damaging free radicals and toxic aldehydes via direct chemical reactions between ozone and ethylene [62]. Ethylene acts as a signaling molecule by binding to one or more receptors, and the above discussion suggested that the ethylene signaling pathways could be activated in response to ozone causing leaf damage.

#### 5.2.3. Jasmonic Acid

Jasmonic acid (JA) is involved in the suppression of ROS-dependent lesion development through the ozone-sensitive JA-deficient mutants in ozone-exposed leaves. In Arabidopsis the JA-insensitive 1 mutants are ozone-sensitive (oji1), coronatine insensitive1 (coi1), methyl jasmonate-resistant 1 (jar1), while 12-oxophytodienoate reductase 3 (opr3) mutants are sensitive to ozone [63]. Previous studies reported that pretreatment with MeJA conferred ozone tolerance on Arabidopsis and tobacco, which indicates an involvement of JA in ozone stress [63]. MeJA induced genes that encoded an ethylene receptor (ERS2), whereas leaf damage was inhibited via these receptors [64]. Ozone caused the development of ethylene-dependent lesions that could be inhibited by JA. The JA-induced genes such as AT2G24850 and AT5G24770 are shown in Table 1. JA might also be involved in the production of antioxidants that protect leaves against damage by ozone [53]. MeJ-treated plants showed gene expression of antioxidants like ascorbic acid (VTC1, VTC2, DHAR, and MDHAR) and glutathione (GSH1 and GSH2) [65]. Thus, it is concluded that the accumulation of antioxidants induced by JA in plants could support increased ozone tolerance.

## 6. Phytohormone Signaling Cross-Talk in Response to Ozone

A number of recent studies revealed that several classical phytohormones (ET, ABA, IAAs, and CKs) and signaling molecules (SA, JA, Pro, and BRs) responded to chronic and acute ozone exposure [7,9]. Ozone exposure induced the generation of ROS in cells leading to PCD, which was regulated by a process of feedback inhibition [66]. In this section, we update the existing knowledge concerning the relationship between phytohormones and signaling intermediates, and their antagonistic and synergistic cross-talk in complex signaling pathways in response to acute ozone stress.

**Figure 3:**
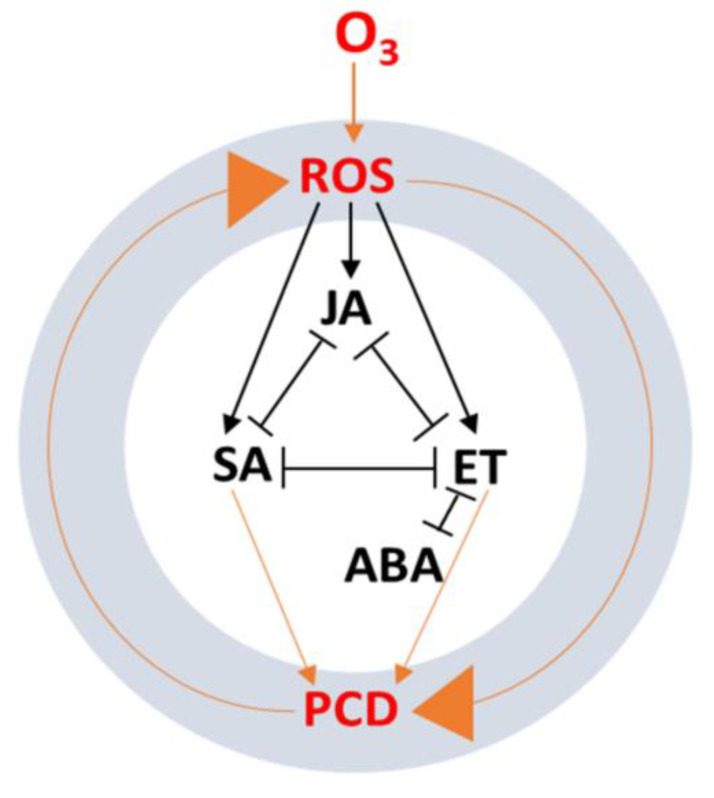
Programmed cell death (PCD) cycle induced by ozone (O_3_) associated with Phytohormones, adapted from Kangasjarvi et al. (2005) [67]. Reactive oxygen species (ROS) accumulation is caused by ozone stress and results in the accumulation of salicylic acid (SA) and PCD. Cell death activates ethylene (ET) production required for ROS generation resulting in PCD. Jasmonic acid (JA) antagonizes cell cycle progression by inducing cell death and promoting SA and ET function. Abscisic acid (ABA) antagonizes the function of ET, which may have an important role in ozone-induced PCD.

Recently, more attention has been given to exploring the interaction of phytohormones in response to ozone stress in plant cells [47]. It has been proposed that ozone stress causes the generation of ROS, resulting in ET, SA, and JA production along with PCD, where ET accumulation is required for continued ROS generation (Figure 3) [67].

In the oxidative cell death process, JA, SA, and ET show antagonistic influences on each other. Another classical phytohormone, ABA, is required for stomatal closure to block ozone entry and also appears to inhibit ET’s activity in accelerating ozone-induced cell death. The induction of ET biosynthesis was observed in plants within a few hours of ozone treatment [68]. The ET synthesis genes, Le-ACS1A, Le-ACS2, and Le-ACS6, were induced in ozone-treated tomatoes, but the expression of Le-ACS6 and -ACS4 and St-ACS5 was unregulated in potato [9]. The transcription of ACS1, ACS2, and ACO1 was significantly enhanced in beech trees in response to long-term ozone exposure [69,70]. ABA plays a pivotal role in plant growth and development along with the regulation of gene expression associated with the stomatal aperture [71]. It has been revealed that ABA is involved in the response to drought stress by stimulating guard cells to close stomata [72]. There is also evidence that ozone-induced ET may be involved in the stomatal response by interacting with ABA in ozone-sensitive species which leads to stomatal opening. Ozone-induced ET accumulation can disrupt ABA-mediated signaling to stomata [31].

It has also been reported that ROS signaling triggers an auxin response through a combined mechanism. In this process, O_3_ was considered as a model ROS inducer in plants, and the transcriptome results suggested that auxin homeostasis and signaling were altered by apoplastic ROS, which modulated gene expression in Arabidopsis [73]. Very few studies have been documented concerning the protective role of CKs in oxidative stress following ozone exposure in several crops. Supplementation with kinetin retarded leaf yellowing controlled the reduction of free sterols and inhibited ozone-induced foliar necrosis of the bean [9].

## 7. Conclusions and Future Perspectives

The significant findings of the past decade in the field of ozone research in plants provide a solid foundation for the widely accepted belief that the degree of ozone exposure affects the regulation of stomata through a coordinated process of signaling, transport, and phytohormonal responses. Variation in ozone exposure induces distinct phytohormone profiles and signaling responses in plant cells. In this review, we have updated the existing knowledge concerning ozone-induced stomatal regulation and the involvement of classical phytohormones in signaling guard cells following ozone exposure in plants. We explored the mechanisms associated with phytohormone-mediated stomatal closure and opening in response to ozone. Moreover, we explored the MAPK signaling pathway in response to ozone in plants. However, the gaps in our understanding mean that it will be necessary to further investigate the unique physiological, molecular, and biochemical networks activated in response to ozone stress. This will provide essential data on (i) the mechanisms underlying divergence in the stomata in response to ozone, (ii) alterations in metabolite levels in guard cells under ozone exposure, (iii) the interactions between guard cell metabolites and plasma membrane proteins, (iv) the O_3_-mediated activation of mitogen-activated protein kinases (MAPK), and (v) the initiation, progression, and localization of cell death caused by O_3_. We believe that continued studies of these mechanisms will create a more detailed picture of the nature of ozone stress in plants and indicate potential strategies for overcoming the negative effects.

## Figures and Tables

**Table 1:** Ozone (O_3_)-induced phytohormone responsive genes in different ecotypes of Arabidopsis (modified and rearranged from Li et al. (2006) [53]).

Gene Accession	Gene Family	Response Marker Gene/Metabolites	Col-0 (Control vs. Stress)	Cvi-0 (Control vs. Stress)	Col 0 and Cvi-0 (Control vs. Stress)
SA induced:					
*AT2G14610*	PR-1-like protein	induced	0	+	−
*AT1G19320*	pathogenesis-related protein 5 precursors,	induced	+	0	+
*AT2G22300*	ET-induced calmodulin-binding	NFkB2-F	0	+	0
ET induced:					
*AT1G49830*	ET-responsive protein ET-responsive element binding	Not responded	0	+	−
*AT1G55150*	box RNA helicase protein, putative ET-responsive element binding	Not responded	0	+	0
*AT2G22300*	binding plant defensin protein, putative	NG_k_B2-F	0	+	0
*AT5G44420*	(PDF1.2a) plant defensin protein, putative	induced	−	−	0
*AT2G26020*	(PDF1.2b)	induced	−	−	0
JA induced:					
*AT2G24850*	putative tyrosine aminotransferase	induced	−	−	+
*AT5G24770*	vegetative storage protein Vsp2	induced	0	0	+

**Table 2:** Ozone (O_3_)-induced antioxidant genes in different ecotypes of Arabidopsis (modified and rearranged from Li et al. (2006) [53]).

Gene Accession	Functional Group	Col 0 (Control vs. Stress)	Cvi-0 (Control vs. Stress)	Col 0 and Cvi- 0 (Control vs. Stress)
*AT3G09640*	ascorbate peroxidase	repressed	stable	induced
*AT2G28190*	thylakoid bound ascorbate peroxidase	repressed	stable	repressed
*AT3G10920*	manganese superoxide dismutase	stable	induced	repressed
*AT1G03850*	glutaredoxin protein family glutaredoxin	stable	induced	repressed
*AT2G41680*	thioredoxin	stable	stable	repressed

**Table 3:** O_3_-induced regulation of genes in wild type and both triple mutants of Arabidopsis plants (adapted from Xu et al. (2015) [55]).

Gene Accession	Gene Name	Response (WT O_3_/WT Control)	Response (coi1 ein2 sid2 O_3_/coi1 ein2 sid2 O_3_ Control)
*AT1G15520*	PDR12	Up-regulated	Stable
*AT1G26380*	FAD-binding Berberine family protein	Up-regulated	Stable
*AT1G21120*	IGMT2	Up-regulated	Stable
*AT2G26560*	PLA2A	Up-regulated	Stable
*AT4G08770*	PRX37	Up-regulated	Up-regulated
*AT4G30280*	XTH18	Up-regulated	Up-regulated
*AT5G65730*	XTH6	Down-regulated	Down-regulated
*AT2G42380*	BZIP34	Down-regulated	Down-regulated
*AT1G60590*	Pectin lyase-like	Down-regulated	Down-regulated
*AT5G15310*	MYB16	Down-regulated	Down-regulated

## Data Availability

Not applicable.
